# Investigating the effects of vaccine on COVID-19 disease propagation using a Bayesian approach

**DOI:** 10.1038/s41598-023-37972-7

**Published:** 2023-08-17

**Authors:** Lu Ling, Satish V. Ukkusuri

**Affiliations:** Lyles School of Civil Engineering, Purdue University, West Lafayette city, 47906 USA

**Keywords:** Diseases, Disease prevention, Health policy, Psychology and behaviour, Socioeconomic scenarios

## Abstract

The causal impact of COVID-19 vaccine coverage on effective reproduction number *R*(*t*) under the disease control measures in the real-world scenario is understudied, making the optimal reopening strategy (e.g., when and which control measures are supposed to be conducted) during the recovery phase difficult to design. In this study, we examine the demographic heterogeneity and time variation of the vaccine effect on disease propagation based on the Bayesian structural time series analysis. Furthermore, we explore the role of non-pharmaceutical interventions (NPIs) and the entrance of the Delta variant of COVID-19 in the vaccine effect for U.S. counties. The analysis highlights several important findings: First, vaccine effects vary among the age-specific population and population densities. The vaccine effect for areas with high population density or core airport hubs is 2 times higher than for areas with low population density. Besides, areas with more older people need a high vaccine coverage to help them against the more contagious variants (e.g., the Delta variant). Second, the business restriction policy and mask requirement are more effective in preventing COVID-19 infections than other NPI measures (e.g., bar closure, gather ban, and restaurant restrictions) for areas with high population density and core airport hubs. Furthermore, the mask requirement consistently amplifies the vaccine effects against disease propagation after the presence of contagious variants. Third, areas with a high percentage of older people are suggested to postpone relaxing the restaurant restriction or gather ban since they amplify the vaccine effect against disease infections. Such empirical insights assist recovery phases of the pandemic in designing more efficient reopening strategies, vaccine prioritization, and allocation policies.

## Introduction

The COVID-19 pandemic has been reported to cause 6 million deaths worldwide since its initial outbreak in 2019 and has deeply disrupted all aspects of daily life^1^. In the absence of vaccines against COVID-19 infection, mitigation of disease propagation was mostly achieved by implementing a wide range of non-pharmaceutical interventions (NPIs) and actions by restricting movement^1–4^ throughout the world, ranging from the emergency declaration, lockdowns, closure of non-essential businesses, social distancing mandates, to the increased testing and tracing. The trade-off between disease prevention and movement restrictions sacrifices the economy and social needs. Indeed, the speed and extent of COVID-19 propagation have challenged the economic system and public health care system of many counties across the world^1,5^. Fortunately, lab experiments proved vaccines efficacy in against COVID-19 infection^6,7^, such as Pfizer-BioNTech, Moderna, or Janssen COVID-19 vaccines. An opportunity for a gradual reintroduction of active business events and mobile population by lifting the NPIs can be achieved based on the availability of vaccines. However, vaccine efficacy varies on the age-specific population^6,7^ and is proved to decay over time^6^. That makes the causal impact of vaccine coverage on disease spread rate in the real-world scenario, measured as the decreased quantity of effective reproduction number *R*(*t*) caused by vaccine coverage, vary in a temporal-spatial granularity. This fact could make the economic recovery process in the U.S. confront numerous obstacles while reestablishing a productive and socially acceptable environment. Indeed, CDC^8,9^ indicated that NPIs are still needed to prevent disease propagation and mortality during the process of increasing vaccine coverage. Besides, Wang et al.^10^ pointed out that prematurely reopening social contacts could initiate a new pandemic in the U.S. if vaccine coverage is low. Hence, quantifying the causal impact of vaccine coverage on *R*(*t*), defined as the vaccine effect in our study, is essential to assist optimal reopening strategies, which is critical to relieving the socio-economic burden in the COVID-19 emergency phase^11^.

Previous studies^6,7^ have analyzed the causal impact of the vaccine on disease infections through lab experiments and a small-scale of targeted people. Although such studies provide a general understanding of the vaccine effect in a controllable environment, they retain two critical drawbacks. First, observations are limited to an ideal setting by ignoring the multiple influencing factors, which introduces biases when adopting them to real-world scenarios. Second, the applied methods fail to model the causal effect of the vaccine in a quantifiable, continuous, and longitudinal manner, which requires a statistical framework that predicts the performances of *R*(*t*) if the vaccine coverage is below the designed value. In real-world scenarios, the difficulty of investigating the causal impact of vaccine coverage is the confounding issue caused by influencing factors of *R*(*t*). Indeed, the larger airport hubs^12^ are an important indicator that produces a high influx of imported infections and boosts the local transmission of the virus. Besides, the local disease dynamics are greatly influenced by factors such as the demographic characteristics^13–15^, the presence of disease variants^7,8^, the protective behavior from imposed policies (e.g., mask requirement)^16–18^, mobility index and social contacts^19–21^.

**Figure 1:**
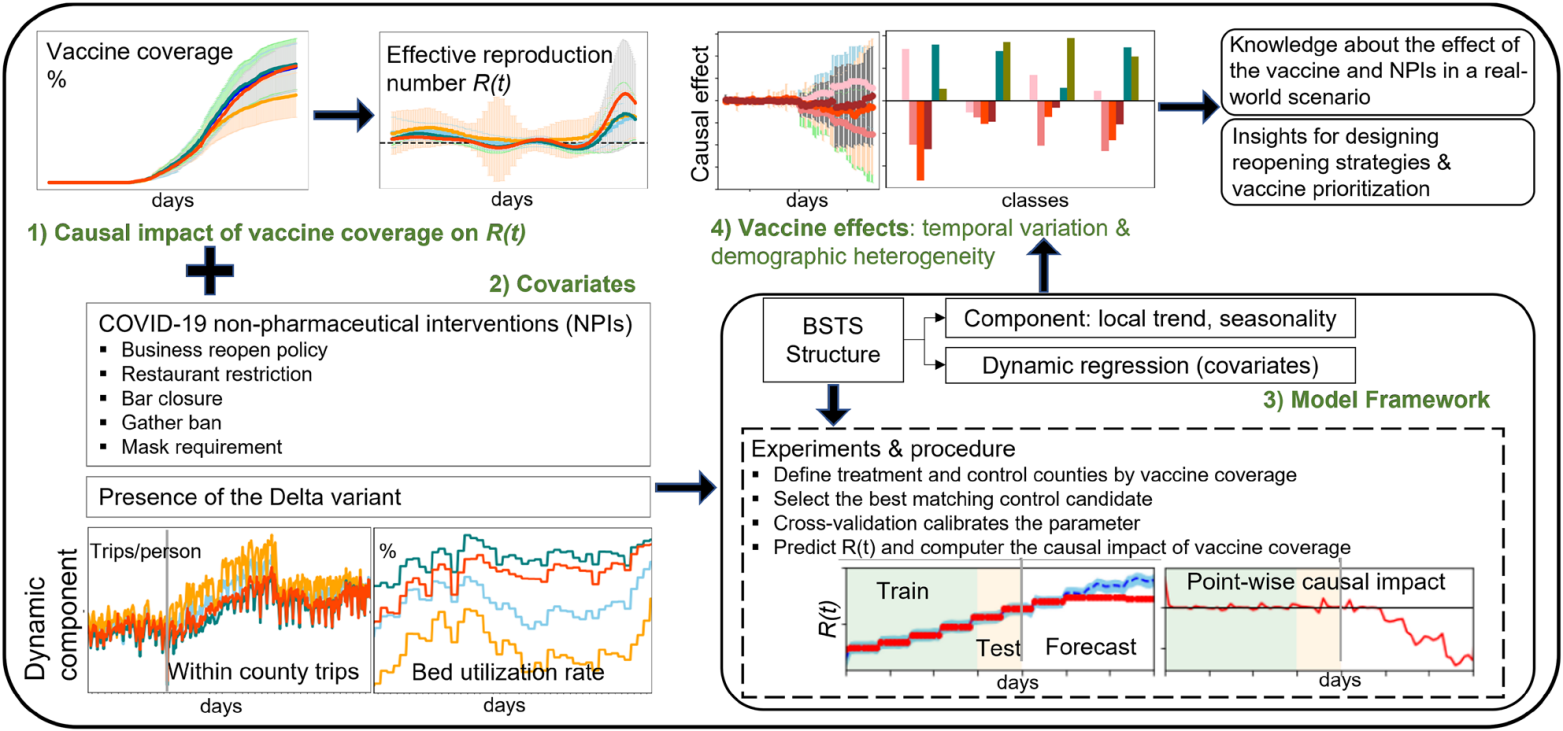
Study overview.

In this study, to overcome the aforementioned issues, we first classify the U.S. counties into four groups based on county-level demographic information (e.g., population density, hospital bed capacity, median household income, and the percentage of older people) and core airport hubs. This procedure specifies the experiment by testing the causal impact of vaccine coverage within each county group. Then, we cut the study period from January to July 2021 into six continued experiments corresponding to vaccine coverage of 0%, 10%, 20%, 30%, 40%, and 50%. That makes the influencing policies much more controllable within a shorter period. Besides, we consider a set of covariates in the model to avoid the confounding issue, such as the NPI measures (e.g., business reopening policy, restaurant restriction, bar closure, gather ban, and mask requirement), the presence of Delta variant, mobility index, and the hospital bed utilization rate. Last but not least, we apply the Bayesian structural time series^22^ (BSTS) model to capture the causal impact over a longitudinal time horizon rather than 2 static points, which is much more flexible than the conventional difference in difference models^23^ and Rubin causal model^24^. The overview of the study is presented in Fig. 1, which contains the objective, input data, model framework, and results. This study aims to shed light on: first, demographic heterogeneity and longitudinal time variation of the causal impact of vaccine coverage on disease propagation; second, the role of NPI measures on the vaccine effect and disease propagation. We propose four corresponding hypotheses to investigate these research questions: (H1) Vaccine coverage has a higher impact on preventing disease spread in counties without core airport hubs than in counties with core airport hubs. (H2) Mask requirement has a higher effect on decreasing disease spread for counties with low population density than counties with high population density. (H3) The vaccine effect for counties relaxing NPIs in the early time is significantly higher than for counties relaxing NPIs later. (H4) Mask requirement amplifies the vaccine effect under the presence of the Delta variant. The exploration of these hypotheses helps to gain knowledge about the effect of the COVID-19 vaccine and the NPIs in a real-world scenario. Furthermore, it assists in the economic recovery process in designing vaccine prioritization and reopening strategies for COVID-19 as well as further pandemics.

## Results

### The causal impact of vaccine coverage on effective reproduction number *R*(*t*) for counties with different demographics

**Figure 2:**
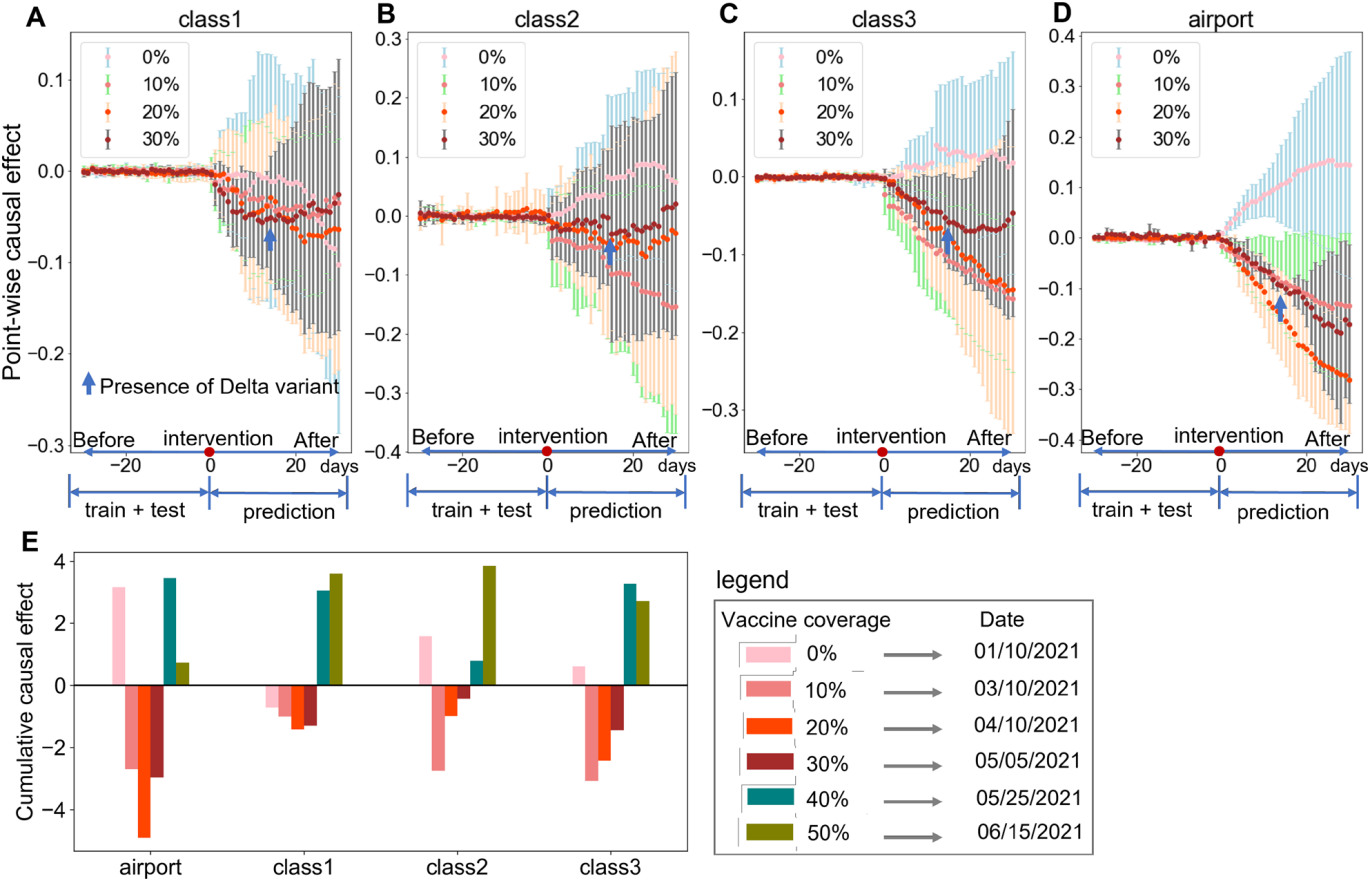
Causal effect of vaccine coverage for counties with different demographics. The demographic characteristics of each county are presented in Table 1. (**A**–**D**) present the point-wise causal effect for each county class. (**E**) shows the cumulative causal effect for each county class.

To investigate the first hypothesis, we aggregate the causal impact of vaccine coverage on *R*(*t*) over the time horizon by county category and coverage level. The mean and standard deviation in Fig. 2A–D characterize the temporal variation of vaccine effect for 30 consecutive days, and the causal impact in Fig. 2E designates the demographic heterogeneity of cumulative vaccine effect for each county class.

Figure 2 E presents the average cumulative causal impact ($\Theta =\sum _t\theta _t, t=1,2,\ldots ,30$) of vaccine coverage at 0%, 10%, 20%, 30%, 40%, and 50%. The cumulative causal impacts are aggregated by county class for each vaccine coverage. The numbers of $\Theta _i (i=1,2,3,4)$ should be interpreted as “the number of effective reproduction number *R*(*t*)-as-usual days worth of impact”. For example, counties with core airport hubs experienced a cumulative median vaccine effect of $\Theta =-2.6$ after 30 consecutive days when vaccine coverage was 10% on March 10, 2021. This suggests that the *R*(*t*) was decreased by 2.6 on 30 consecutive days for counties with core airport hubs compared with the *R*(*t*) if the vaccine coverage was above 10% on March 10, 2021. Most counties experienced a positive impact at vaccine coverage of 0%, except for counties (“class1”) with high median income, low population density, low percentage of older people, and low hospital bed capacity. The vaccine does not immediately reduce *R*(*t*). That might be because that imposed control measures were gradually relaxed after January 2021, and people’s less cautious about maintaining protective behaviors as soon as they have the injection^25^ led to a surge in infection risk. We also clearly observe the demographic disparity in the negative causal impact of vaccine coverage across county classes from vaccine coverage of 10% to 30%. In particular, the significantly higher vaccine effect for counties with core airport hubs ($-3.8$) than counties without core airport hubs on average with a t statistic of 73 at 0.05 significance level leads to the rejection of H1. However, the cumulative negative effects gradually shift to positive as vaccine coverage reaches 40% and 50% for all county classes after May 25, 2021, which seems a counter-intuitive observation. The presence of the Delta variant at the end of April 2021 (when vaccine coverage reaches 40%) might account for that observation since it leads to the surge of infection risk and decreases the vaccine effect when it initially spreads, which parallels the previous study^7^.

To further understand the impact of vaccine coverage over a longitudinal time horizon, we summarize the mean and standard deviation of the point-wise causal impact of vaccine coverage in Fig. 2A–D. Each dot in Fig. 2A–D corresponds to the difference between the actual and the predicted *R*(*t*) across time ($\theta _t= \hat{R(t)}-R(t)$) in vaccine coverage of 0%, 10%, 20%, and 30%. The vertical error bars show the standard deviation of the estimated causal effect. The negative value of $\theta _t$ means that the vaccine had a negative impact on *R*(*t*), resulting in the decrease of *R*(*t*) and otherwise. Several interesting observations can be made from these visualizations. First, we notice that the impact of vaccine coverage on *R*(*t*) is not linear. The age-structured vaccine allocation strategy (e.g., the first got vaccinated age group) might be a potential reason for that observation as indicated by Matrajt et al.^26^. Second, we see the negative vaccine effect decreases earlier for counties (“class2”) with a high percentage of older people, low population density, low median household income, and low hospital bed capacity (starting at vaccine coverage of 20%) than it for other county classes (start at vaccine coverage of 30%). The surge of infection risk introduced by the Delta variant at the end of April 2021 accounts for the decreased negative vaccine effect^7^. It also indicates that older people are more vulnerable to being infected by the new variant than others. This finding is consistent with Powels et al.^7^. Third, from the cross-comparison of vaccine effects for county classes, the average point-wise vaccine effect for counties with core airport hubs is higher than for other counties. A potential reason could be that people who plan for long travel are more cautious about mask-wearing, which might amplify the vaccine effect by interfering with the virus transmission process^17,27^.

### Role of NPI measures on effective reproduction number *R*(*t*)

**Figure 3:**
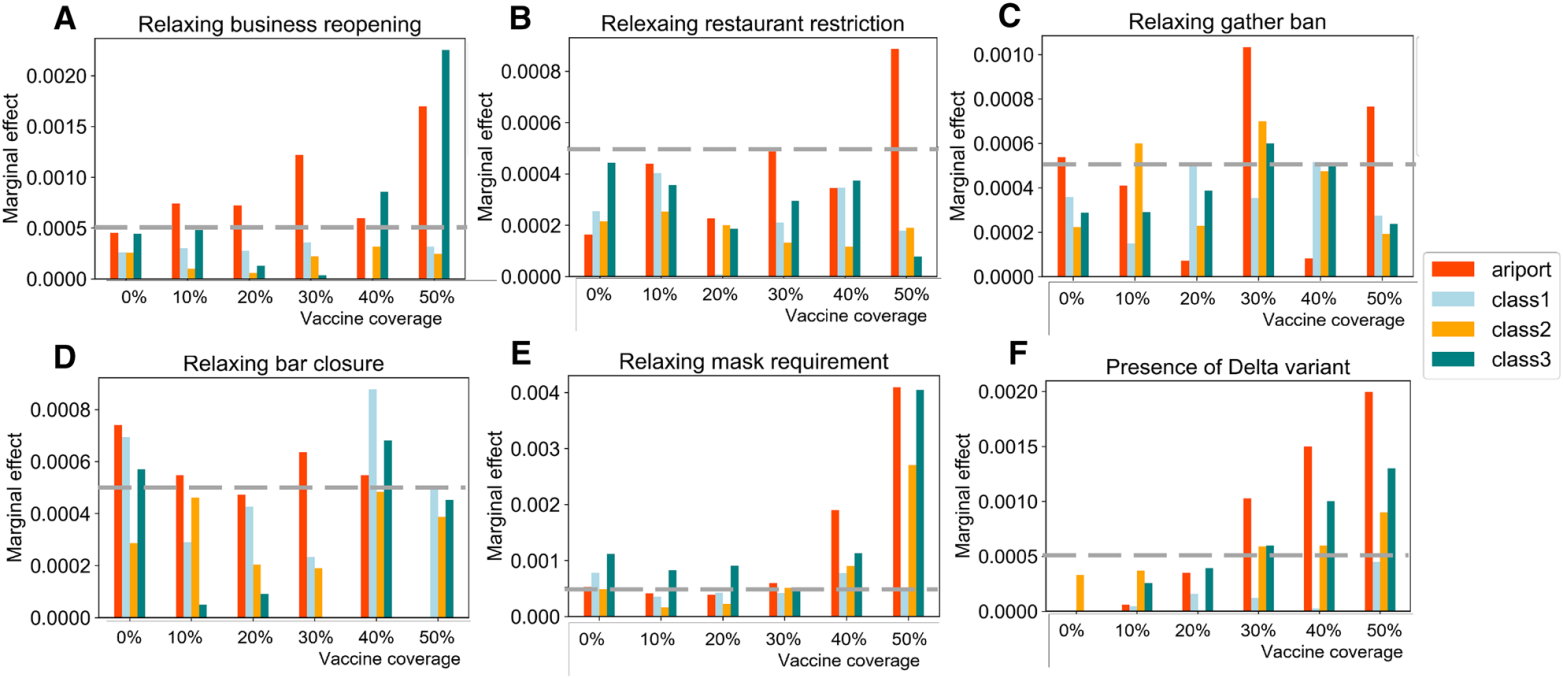
Average marginal effect of policies on disease infections. The demographic characteristics of each county class are presented in Table 1. (**A**–**F**) summarizes the marginal effects of studied NPIs on *R*(*t*) at each designed vaccine coverage.

To examine the second hypothesis, we regress NPI measures (e.g., business reopening, restaurant restriction, gather ban, bar closure, and mask requirement) as covariates in a dynamic approach when investigating the vaccine effect. We measure the mean effect of NPIs by calculating the mean of marginal effects in each county and aggregating them based on the county class. Figure 3 summarizes the continuous and longitudinal effect of the NPIs and the presence of the Delta variant on disease infections for each county class at each vaccine coverage.

The interpretation of the positive marginal effect of NPI measures should be “the increased number of *R*(*t*) by relaxing one step of the NPI measure”. And the steps within each NPI measure are presented in Table 2. For example, Fig. 3 A shows that relaxing one step of the business reopening policy (e.g., from “restrictions” to “easing restriction”) increases 0.0022 of *R*(*t*) for counties (“class3”) with high population density, high median income, high hospital bed capacity, and low percentage of older people at vaccine coverage of 50%. For the impact of NPI measures on *R*(*t*), we gain three observations from Fig. 3A–D. First, we see that the marginal effect of relaxing the business reopening policy for high population density counties (“class3”) is about 3.5 times that of counties with low population density (“class1” and “class2”). That indicates high population density counties have a higher infection risk by relaxing business reopening policy due to their active commercial activities. Second, we observe a lower marginal effect of relaxing restaurant restriction, gather ban, and bar closure policies on average compared with the marginal effect of relaxing business reopening policy for all counties classes in Fig. 3B–D. Third, We observe that the effects of relaxing mask requirement in Fig. 3E increase along with augmenting vaccine coverage overtime for all counties. In particular, a significantly higher marginal effect (t statistic of 93 at 0.05 significance level ) for counties with core airport hubs (“airport”) or high population densities (“class3”) than for counties with relatively low population density (“class1” and “class2”) leads to the rejection of H2. It indicates that mask requirement is more effective in decreasing disease infection for counties with core airport hubs or high population density. Indeed, Barasheed et al.^28^ suggested that wearing masks in crowded places could reduce the risk of respiratory infections by 20%. More importantly, although the presence of the Delta variant significantly increases the infection risk as described in Fig. 3F, our results indicate that the mask requirement is more effective than other NPIs in preventing the disease spread after 50% people got vaccinated, which parallels the finding suggested by Brüssow and Zuber^17^.

### Role of postponing relaxing NPI measures on vaccine effects

**Figure 4:**
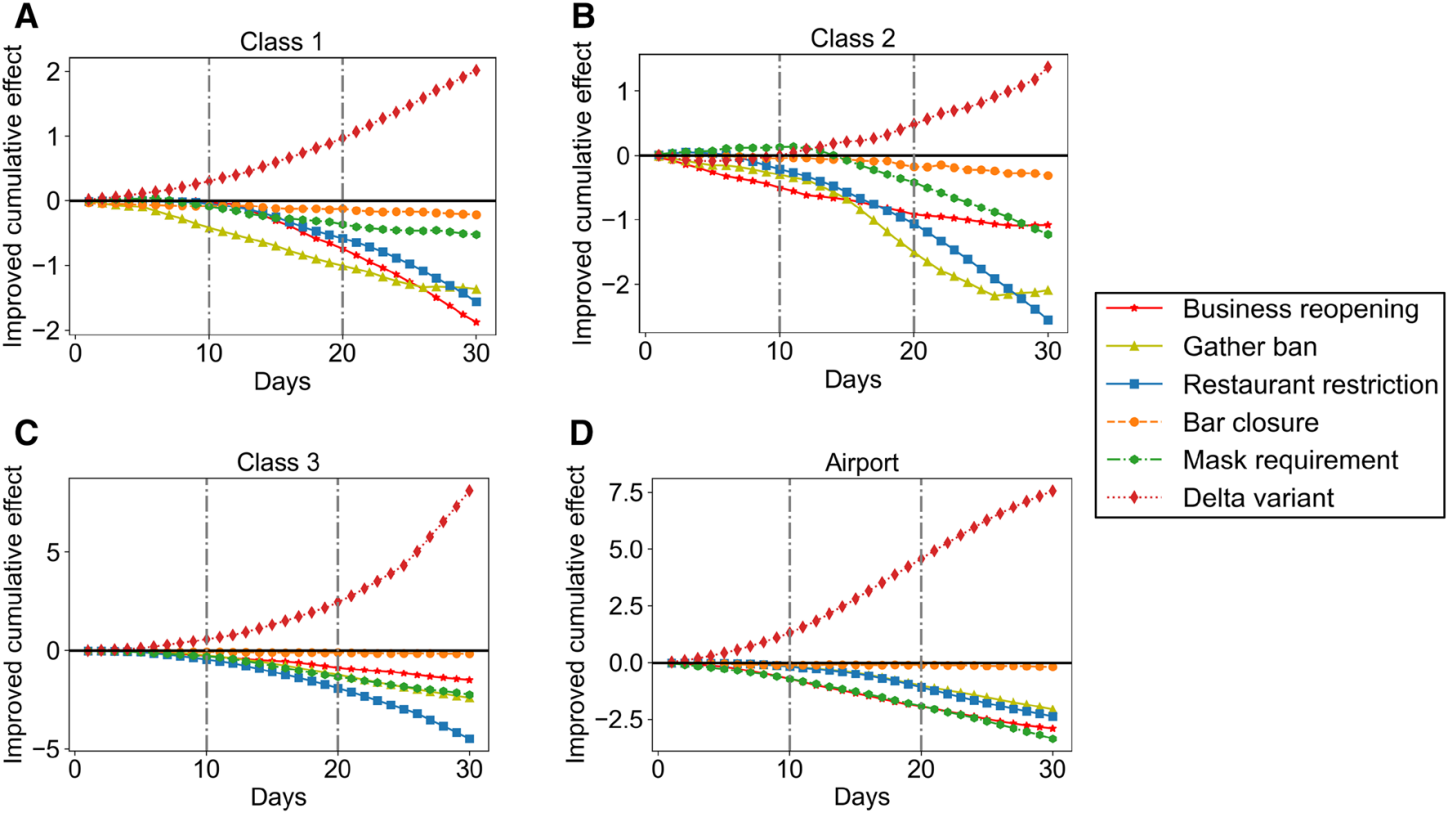
Improved vaccine cumulative effect on disease propagation for applying different policies 15 days earlier versus 15 days later. The demographic characteristics of each county class are presented in Table 1.

To examine the third hypothesis, we selected two groups of counties *A* and *B*, within the same county class. Group *A* contains counties applying the NPIs 15 days earlier than counties within group *B* during the study period. Then, we used the $\phi$ to measure the role of NPIs on vaccine effects, and it is calculated by using the average cumulative vaccine effect of group *A* to subtract it from group *B* such that $\phi =\overline{\sum \sum _{i,f\in A}\Theta _{i,T,f}}-\overline{\sum \sum _{i,e \in B}\Theta _{i,T,e}}$, where $\phi$ is the cumulative causal effect. *f* and *e* belong to group *A* and *B* separately and are counties within the same county class at designed vaccine coverage *i*. *T* is the observation period and is defined as 30 days. Figure 4 shows the improved cumulative vaccine effect $\phi$ for each policy. The negative values of $\phi$ mean that the vaccine could decrease more number of *R*(*t*) by postponing relaxing the control policy for 15 days, otherwise. For example, Fig. 4A indicates that the vaccine could reduce the *R*(*t*) by 2 more if counties (with high median household income, relatively low population density, low percentage of older people, and low hospital bed capacity) postponed relaxing the business reopening policy by 15 days ($\phi =-2$ for postponing relaxing business reopening policy).

From Fig. 4A–D, we observe the disparity of the role of NPI measures in the vaccine effect across county classes. Several interesting observations can be made from these visualizations. First, we notice that postponing relaxing the restaurant restriction amplifies the vaccine effect of reducing *R*(*t*) for all counties, while postponing relaxing the bar closure barely affects the vaccine effect. This observation leads to the rejection of H3. Second, postponing relaxing mask requirement amplifies the vaccine effect by 2 more times for counties with core airport hubs (“airport”) and counties (“class3”) with high population density, high median household income, high hospital bed capacity, and low percentage of older people than counties with low population densities (“class1” and “class2”). Third, in addition to restaurant restriction, postponing relaxing gather ban can also significantly amplify the vaccine effect of reducing *R*(*t*) for counties (“class2”) with a relatively high percentage of older people, low population density, low median household income, and low hospital bed capacity. Last but not least, we observe that the presence of the Delta variant significantly decreases the vaccine effect of reducing *R*(*t*) for all counties, especially the impact of the presence of the Delta variant for counties with core airport hubs (“airport”) is significantly higher than its effect for counties without core airport hubs (“class1”, “class2”, and “class3”). That finding is consistent with our previous statement that the causal impact of vaccine coverage is lower for counties with core airport hubs than counties without core airport hubs after the presence of the Delta variant. That might be because core airport hubs connecting cross-border mobility and gathering it into a high spatial density movement results in high infection risk among people^12^.

### The Role of mask requirement on the vaccine effect with the presence of Delta variant

**Figure 5:**
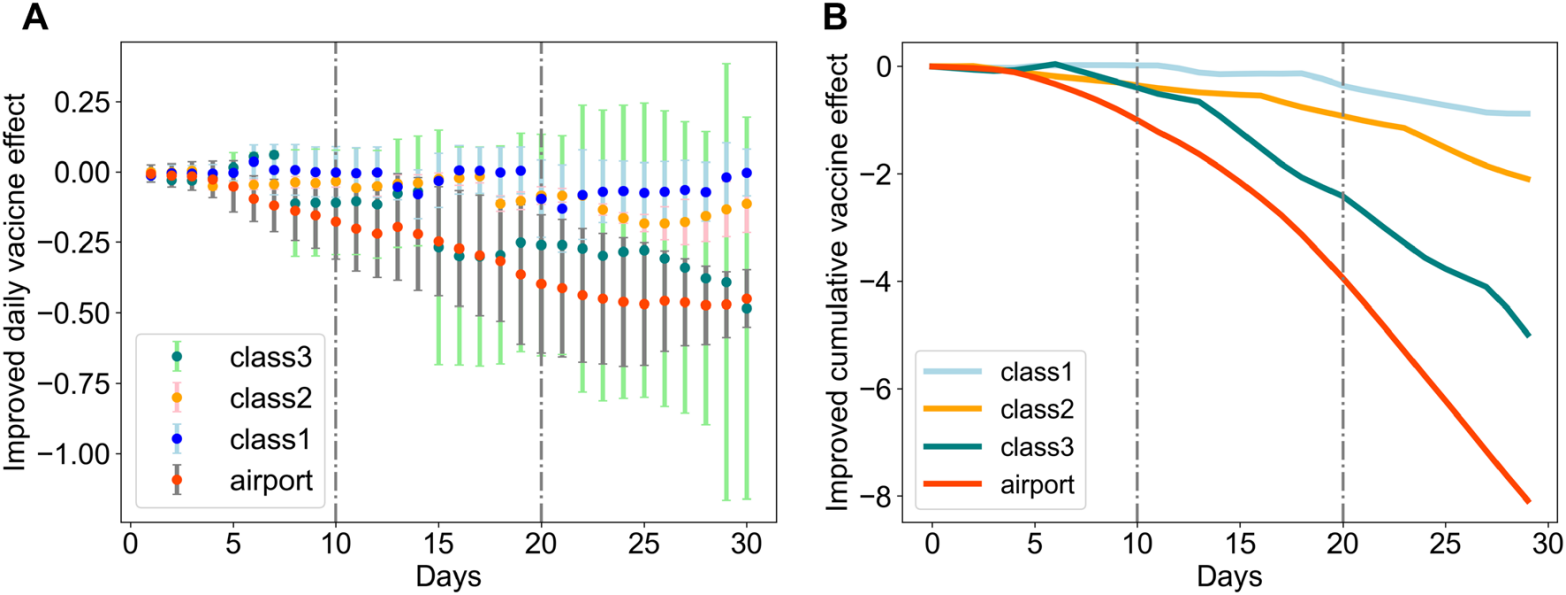
Improved effect of the vaccine on disease propagation under the presence of Delta variant (with vs. without mask requirement). The daily vaccine effect is calculated by using the point-wise vaccine effect under the presence of the Delta variant for counties with mask requirements to subtract the point-wise causal effect of vaccine for counties without mask requirements. The demographic characteristics of each county class are presented in Table 1.

Mask wearing has been highly recommended by CDC^16^ due to being less costly to apply than other NPI measures, which are associated with high social costs^17^. However, the role of mask requirement on vaccine effect after the Delta variant remains to be quantified. The fourth hypothesis aims to gain insight into whether the mask requirement is needed when the vaccine coverage for the general public reaches 50%, which corresponds with the presence of the Delta variant. To construct the experiment, we selected two groups of counties *C* and *D* within the same county class, with group *C* keeping applying the mask requirement and group *D* relaxing the mask requirement after the vaccine coverage reaches 50%. We quantify improved effects by taking the difference of vaccine effects (both daily and cumulative) for two counties groups ($\sigma _t=\theta _{C,t}-\theta _{D,t}$ and $\Sigma =\sum _t \sigma _t, t=1,2,\ldots ,30$). Figure 5 A summarizes the average improved daily effect, and Fig. 5B introduces the cumulative effect for each county class. Negative values of $\sigma _t$ and $\Sigma$ would mean that the vaccine would reduce more number of *R*(*t*) by applying the mask requirement policy after the presence of the Delta variant. For example, the vaccine would reduce 0.5 ($\sigma _{30}=-0.5$) more of the *R*(*t*) on the day of 30 if counties with core airport hubs apply the mask requirement than those without the mask requirement after the presence of the Delta variant.

Figure 5 presents a demographic heterogeneity in the role of mask requirement on vaccine effect across counties. We observe that there is a significantly improved daily negative vaccine effect by applying mask requirement after the presence of Delta variant for almost all counties (t statistic being 65 and 109) except the counties (“class1”) with a high median household income, low percentage of older people, low population density, and low hospital bed capacity. That observation leads to the rejection of H4. Besides, we observe that the improved negative vaccine effect is about 2 times higher after applying the mask requirement for counties with core airport hubs than counties without core airport hubs. In particular, we see that counties with high population density, high median household income, high hospital bed capacity, and a low percentage of older people obtain the highest improved negative vaccine effect among counties without core airport hubs. It indicates that the mask requirement amplifies the vaccine effect of reducing disease propagation for counties with high mobility and activities needs. In conclusion, the estimated results suggest that although the mask requirement does not amplify the vaccine effect for all areas, it greatly amplifies the vaccine effect in reducing the viral burden against the disease infection for areas with core airport hubs, high population density, and vulnerable people even with the presence of the Delta variant. Palcu et al.^18^ also suggested that facial masks should be encouraged even after the vaccine is available.

## Discussion

Although increasing vaccine coverage is the most effective approach against COVID-19 infections, the optimal reopening strategy is still needed to balance the trade-off between disease prevention and economic recovery in the short term. However, the lack of understanding of the causal impact of dynamic vaccine coverage and the joint effects of NPIs in real-world scenarios limits us in designing the optimal vaccine prioritization and the corresponding reopening strategies. This study examines the vaccine effect’s temporal variations and demographic heterogeneity for U.S. counties based on the causal time series analysis. Furthermore, we examine the role of NPI measures and the presence of the Delta variant on the vaccine effect and disease spread, which allows us to monitor and understand the impact of relaxing imposed policies in the COVID-19 period with unprecedented spatiotemporal granularity and scale. The analysis concludes that (1) The vaccine allocation strategy (e.g., age-specific allocation) would greatly affect the causal impact of vaccine coverage on disease infections. Although vaccine effect in reducing the disease infections for areas with high population density and fewer older people or core airport hubs is 2 times higher than areas with less population density. Areas with more older people need a high vaccine coverage to help them against the more contagious variants (e.g., the Delta variant) due to their physical weakness. Therefore, age and population density-specific vaccine prioritization is needed, especially when facing vaccine supply constraints. (2) Although business restriction policy and mask requirements are proved to be more effective measures to prevent COVID-19 infections than other NPIs for areas with high population density and core airport hubs after the vaccine is available. The mask requirement is more applicable to assist disease prevention during the economic recovery process for two reasons. First, the mask requirement policy is less costly. Second, the mask requirement is more effective than other NPI measures in amplifying the vaccine effects against disease infections for areas with high population density or core airport hubs. In particular, the vaccine would reduce 8 more of the *R*(*t*) in 30 consecutive days by applying the mask requirement for counties with airport hubs after more contagious variants show up. (3) Areas with a high percentage of older people are suggested to postpone relaxing the restaurant restriction or gather ban since these two measures significantly amplify the vaccine effect against disease infections. However, the bar closure policy barely affects the disease infections as well as the vaccine effect. The analysis specifies vaccine effects for counties with different demographics and explores how the NPI measures affect the vaccine effects and disease propagation in the COVID-19 period. The implication assists the economic recovery process in designing a more efficient reopening strategy, vaccine prioritization, and relocation policies.

This study investigates the causal impact of vaccine coverage on *R*(*t*) from a Bayesian time series approach. It has certain limitations related to the causal experiment for the observational study. First, to avoid potential confounding issues in this study, we specify the experiment to a more controllable setting by separating the study period into six continued periods. Besides, we consider NPI measures, the presence of the Delta variant, daily mobility index, and dynamic hospital bed utilization rate as covariates to eliminate the confounding issue in examining the vaccine effects on disease spread. Although the influencing factors of disease infection might not be fully captured in real-world scenarios, we address this issue by matching the most similar control county, which provides a best prior for the unknown factors. Second, additional disease variant shocks (e.g., the Delta variant), present later in the study period, significantly affect the *R*(*t*) value and directly introduce biases in estimating vaccine effects under the lack of the measure of these new variants. We address the interference of the Delta variant by coding it as a binary indicator after it was detected at the end of April 2021. However, it might not be sufficient to capture the full effect later due to its rapid growth. Last but not least, the lack of disaggregated individual-level behavior analysis leaves the underlying reason for our insight to remain to be explored.

Although this study used aggregated measures to quantify the county-level demographic heterogeneity and temporal variation of vaccine effects and the corresponding NPIs at the COVID-19 phase, more microscopic analysis is worth to be explored with additional data. Further research focuses on extending the causal effects of vaccines to a more disaggregated level, where different patterns of vaccine effects on disease propagation can be obtained, and individuals’ activities can be explored to provide a detailed explanation for the vaccine effect. Besides, comparative analysis containing multiple communities could yield novel and more generalizable insights on vaccine effects and the corresponding control measures for developing better disease surveillance and economic recovery strategy. Furthermore, examining vaccine effects on disease spread after the entrance of new disease variants could strengthen the understanding of the reliability of vaccine effects in adjusting to diverse variants under real-world scenarios.

## Materials and methods

### Experiment design

**Figure 6:**
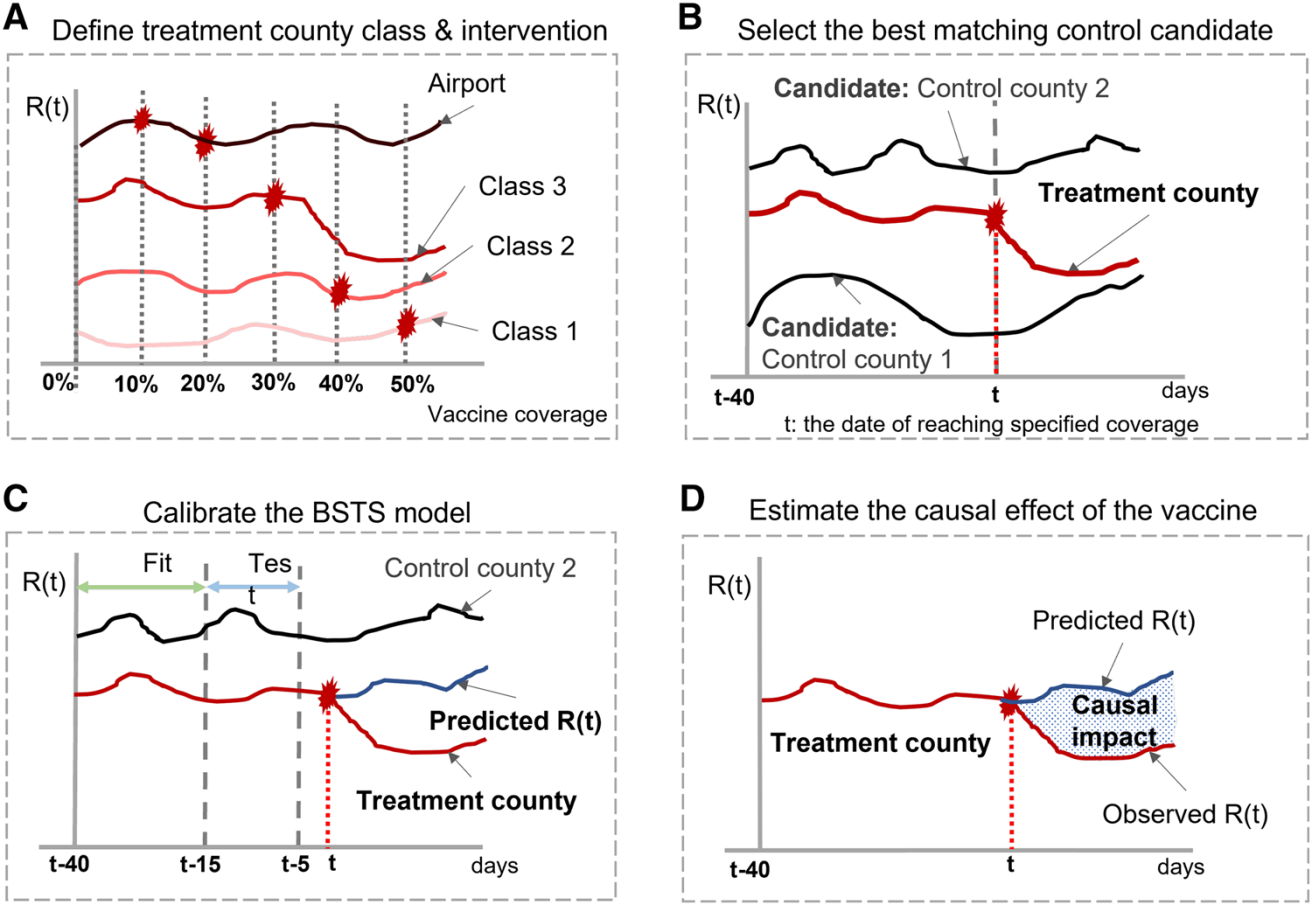
Model procedure. The model procedure follows the order below. (**A**) we define treatment county class and intervention; **B** we select the best matching control candidate; **C** we calibrate the BSTS model; **D** we estimate the causal effect of the vaccine. The date for each designed vaccine coverage are: 0%—01/10/2021, 10%—03/10/2021, 20%—04/10/2021, 30%—05/05/2021, 40%—05/25/2021, 50%—06/15/2021.

The *R*(*t*) is a key epidemic factor in measuring the disease propagation over time. It is the expected number of new infections caused by an infectious individual in a population. The goal of the experiment is to explore the causal impact of the vaccine coverage ( from 0%, 10%, 20%, 30%, 40%, to 50%) on *R*(*t*) and the effects of diverse NPIs on *R*(*t*). NPIs are a set of strategies to prevent disease propagation. In our study, NPIs include business reopening policy, restaurant restriction, bar closure, gather ban, and mask requirements. Within each vaccine coverage level, we conduct one experiment to examine the causal impact. Figure 6 described four steps in each experiment: (1) We classify four class counties by characterizing the demographic heterogeneity of disease dynamics based on K-mean clustering. Within each county class, we cut the time period from January to July 2021 into six continued periods and match each time period with a vaccine coverage level (such as 0%—01/10/2021, 10%—03/10/2021, 20%—04/10/2021, 30%—05/05/2021, 40%—05/25/2021, 50%—06/15/2021). Figure 6 A presents the matching mechanism for each coverage level. That procedure specifies the time period for each experiment to explore the temporal variation of the causal impact. (2) As shown in Fig. 6B, we selected the “treatment counties” and “control counties” within each county class. The “treatment county” is defined by the counties whose vaccine coverage is greater than the designed vaccine coverage level within the time period. Then, we used the time-varying variable *R*(*t*) as the matching similarity metric to select the best matching control county *g*. To avoid the multicollinarity issue, we do not consider other variables in the matching procedure. (3) In Fig. 6C, we predicted the disease propagation *R*(*t*) after the vaccine coverage of the treatment county *f* reaching the designed vaccine coverage *i* (what if the vaccine coverage does not reach the designed coverage?). To avoid the confounding issue, we consider a set of covariates in the prediction including the *R*(*t*) from the best matching control county and non-epidemiological factors^14^. (4) Fig. 6D describes the calculation of the counterfactual disease propagation *R*(*t*) causal impact. We computed the causal impact of the vaccine at the designed coverage *i* by taking the difference between the predicted and observed *R*(*t*) for county *f*.

**Table 1:** The performance of mean scores for each county class after the normalization. “class 1” refers to counties with high median household income, relatively low population density, low percentage of older people, and low hospital bed capacity; “class 2” refers to counties with relatively high percentage of older people, low population density, low median household income, and low hospital bed capacity; “class 3” refers to counties with high population density, high median income, high hospital capacity, and low percentage of older people.

Name	Population density	Median household income	Percentage of the older people	Hospital bed capacity	Core airport hubs	Number
Class 1	0.12	0.56	0.12	0.12	No	97
Class 2	0.02	0.21	0.30	0.08	No	160
Class 3	0.42	0.42	0.13	0.43	No	68
Airport	–	–	–	–	Yes	16

*Note* (1) “airport” refers to counties having core airport hubs in U.S.^29^. (2) older people mean people who are at least 65 years old.

In the first step, we classify counties into four classes based on the critical influencing factors^14,30^ such as population density, the percentage of older people, hospital resources, income, and core airport hubs. The first county class comprises counties with core airport hubs in the U.S. The rest of the county classes are obtained using K-mean clustering^31^ based on population density, median household income, percentage of older people, and the average daily number of beds in the hospital. They are grouped into three classes by comparing the Akaike Information Criterion based on the elbow method. The performance of each county group is presented in Table 1. In the second step, we define the experiment period as 70 consecutive days for each treatment county *f* at designed vaccine coverage *i*. As indicated in Fig. 6B, we select the best matching control county based on the similarity of *R*(*t*) between treatment county and the control county before the date of reaching the designed vaccine coverage (*t*). *R*(*t*) offers a direct observation in timer-varying disease evolution for the control county and the treatment county. In the third step, we calibrate the model parameter via a cross-validation method for the first 40 consecutive days in training and testing periods to ensure the model’s reliability, which is indicated in Fig. 6C. The well-trained model is then used to predict the *R*(*t*) in 30 consecutive days after reaching the designed vaccine coverage. In particular, we consider a set of dynamic covariates in modeling to avoid the confounding issue. In addition to the *R*(*t*) itself, we consider the impact of non-epidemiologic factors on the *R*(*t*), such as diverse NPIs (including business reopen policy, restaurant restriction, bar closure, gather ban, and mask requirement), the presence of the Delta variant, within county trips, and the bed utilization rate. In the fourth step, we define the point-wise causal effect of vaccine at its designed coverage *i* in time *t* for treatment county *f* as $\theta _{i,t,f}=\hat{R(t)}_{i,t,f}-R(t)_{i,t,f}$ and the cumulative causal effect of vaccine at its designed coverage *i* from time $t=0$ to time $t=T$ for treatment county *f* as $\Theta _{i,T,f}=\sum _{t=0}^T\theta _{i,t,f}$. $\hat{R(t)}_{i,t,f}$ is the estimated value by model prediction and *R*(*t*) is the observed value. Then, the key question is how to estimate $\theta _{i,t,f}$.

There are many methods to estimate the causal effect of diverse interventions on the outcome of interest^23,24,32^. Compared to the traditional difference-in-difference model, the structural time series model extensively relaxes the parallel trends assumption and captures the time-varying effects of the outcome of interest. In addition, the Bayesian approach has been widely used to estimate the uncertainty of the parameters in the structure models^33,34^. The BSTS models have been conducted to strengthen causal inference for the time series data by simulating the counterfactual as the synthetic post-treatment controls via posterior predictive samples^20,35^. The BSTS models have been extensively applied in diverse areas such as political science^36^ and socio-economics^35,37^. We used the BSTS model to predict *R*(*t*) for selected treatment counties. BSTS model captures time-varying local trends and seasonality variations for the time-correlated disease spread rate. It enables us to analyze the dynamic effects of vaccines on disease propagation for 30 consecutive days after reaching each designed coverage. The BSTS model for the treatment county *f* is defined as the following^35^:

1 $$\begin{aligned} {\left\{ \begin{array}{ll} y_{t}=Z_{t,}^\intercal \alpha _{t}+\varepsilon _{t}\\ \mu _{t+1}=\mu _{t}+\eta _{\mu ,t}\\ \gamma _{t+1}=-\sum _{s=0}^{s-2}\gamma _{t-s,}+\eta _{\gamma , t}\\ x_t^\intercal \beta _t=\sum _{j=1}^Jx_{j,t}\beta _{j,t}\\ \end{array}\right. } \end{aligned}$$

$y_t$ is the observed *R*(*t*) for the treatment county *f* on day *t* and $\alpha _t$ denoting the unobserved latent states. The evolution of unobserved latent states is defined as $\alpha _{t+1}=T_t\alpha _t+R_t\eta _t$. Matrices $Z_t, T_t$, and $R_t$ contain unknown parameters. The vector $\alpha _t$ is formed by concatenating individual state components, such as the local trend $\mu _t$, seasonality $\gamma _t$, and regression $x_t^\intercal \beta _t$ components. $\mu _t$ represents local variations of the time series data and $\gamma _t$ captures the seasonal variation (e.g., week) with *S* period. $x_t^\intercal \beta _t$ is the dynamic regression components with the coefficient $\beta _{j,t}$ for covariate *j* at day *t* with the time-varying way of $\beta _{j,t+1}=\beta _{j,t}+\eta _{\beta ,j,t}$. Then, the effects of the covariates *j* are estimated from the coefficient $\beta _{j,t}$. A weakly informative prior (Cauchy distribution), which is restricted to a positive number and allows for potentially larger standard deviations^38^, is elicited for each state component. Model parameters of the hyperprior distribution such as $\varepsilon _t,\eta _{\mu ,t}, \eta _{\gamma , t},\eta _{\beta ,j,t}$ are the hyperparameters contained in the BSTS model. They are calibrated using cross-validation method, and 40 consecutive days before reaching the designed vaccine coverage are used to train and test the model. The model training process is presented in Supplementary Fig. 1. Besides, Supplementary Fig. 2A shows the sensitivity of model parameters of the hyperprior. It indicates that the model performance is not sensitive to the selection of hyperparameters (from 1 to 5). Thus, we choose the hyperprior as $\varepsilon _t,\eta _{\mu ,t}, \eta _{\gamma , t},\eta _{\beta ,j,t} \sim Cauchy(0,2.5)$. Besides, we use the estimated residuals to test the normality assumption of the model. The estimated results are presented in Supplementary Fig. 2B. It shows the alignment of the empirical quantiles with the theoretical quantiles.

### Data collection

The experiment resolution is based on the U.S. county-level from October 2020 to July 2021 on a daily basis. We use the county-level *R*(*t*) to measure the dynamic disease propagation and take vaccine coverage as the treatment action of preventing disease propagation. To capture the demographic heterogeneity of the vaccine effect, we classify all selected counties into four classes. The first county class includes counties with core airport hubs, defined by Federal Aviation Administration (FAA)^29^. The rest of the three county classes are classified by the county-level demographic variables obtained from the U.S. Census Bureau’s MAF/TIGER Geodatabases^39^ (e.g., population density, median household income, percentage of older people), and county-level average daily number of beds in the hospital obtained from the Health and Human Service Protect Public Data Hub^40^. In light of the confounding issue, we consider a set of covariates in the causal experiment such as the NPIs obtained from Kaiser Family Foundation^41^, the presence of the Delta variant obtained from local news, daily bed utilization rate obtained from the Health and Human Service Protect Public Data Hub^40^, and the number of trips per person obtained from the U.S. Department of Transportation^42^. To ensure that the disease dynamics are statistically meaningful, we remove counties with incomplete data, which results in 341 counties from 42 states in the study. These counties are classified into four groups based on the demographic variables. The performance of each county class can be found in Table 1. Then, we use selected data to explore the causal impact of the vaccine coverage and the joint effects of covariates on COVID-19 propagation in the U.S.

#### Effective reproduction number *R*(*t*)

The effective reproduction number *R*(*t*) is used to characterize the potential for COVID-19 spread rate at a specific time, defined as the average number of secondary infectious cases generated by a primary infectious case. As indicated by previous study^43^, the virus would spread out if $R(t) > 1$, and the virus would spread locally if $R(t) = 1$, and the virus would stop spreading, and the disease disappears eventually if $R(t) < 1$. We collected the *R*(*t*) from October 2020 to July 2021 from a well-established online resource^44^. Supplementary Fig. 3A shows the variation of average daily *R*(*t*) within each county class. We report that the mean *R*(*t*) value is 1.20, with the standard deviation being 0.51 during the study period.

#### Vaccine coverage

U.S. county-level vaccine data are obtained from the CDC^45^. The vaccine coverage refers to the percentage of fully vaccinated people, which is the county-level percent of people who have a second dose of a two-dose vaccine (Pfizer-BioNTech and Moderna vaccines) or one dose of a single-dose vaccine (Johnson & Johnson’s Janssen vaccine) as reported by CDC. Supplementary Fig. 3B shows the variation in average daily vaccine coverage within each county class. We report that the vaccine coverage ranges from 0 to 69.7% during the study period, with a mean value of 14.7%.

#### NPI measures and the Delta variant

The NPI measures in our study are obtained from Kaiser Family Foundation^41^ including business reopening policy, restaurant restriction, gather ban, bar closure, and mask requirement. Each measure contains multiple steps to match the recovery stages and is presented in Table 2. To capture the effect of the presence of the Delta variant, We collect the earliest state-level detected date of the Delta variant from Web news by hand and apply a 2-weeks delay for an incubation time. Besides, we code the presence of the Delta variant as a binary variable. We visualize the mask requirement, business reopening policy, and Delta detection in Supplementary Fig. 4.

**Table 2:** Status for NPI measures in the COVID-19 period.

Policy	Description
Business reopening	Reopened: 0; Easing restrictions: 1; Restrictions: 2
Mask requirement	No: 0; Unvaccinated people only: 1; Yes: 2
Bar closure	Open: 0; Open with service limit: 1 ; Closed: 2
Gather ban	No limit: 0; Limit on large gatherings: 1; All gatherings prohibited: 2
Restaurant restriction	Open: 0; Open with limited number of people to indoor service: 1 ; Closed to indoor service: 2

#### Bed utilization rate and mobility index

Daily bed utilization rate and individual mobility directly reflect local disease dynamics. In this study, the hospital resource data are obtained from the Health and Human Service Protect Public Data Hub^40^. We calculate the bed utilization rate as the total number of staffed inpatient and outpatient beds divided by the total number of staffed inpatient beds occupied in the hospital during the 7 days. We report that the mean and standard deviation of the county-level bed utilization rate is 60.23% and 16.91%. Previous studies^46,47^ have asserted a close association between mobility and social distancing policies and disease propagation before the vaccine is available. However, this relationship might be changed since people might loosen their protective action based on their vaccinated statues^25^. In this study, we use the county-level daily trips generated by each person to capture the temporal variation of the mobility index. The data are obtained from the U.S. Department of Transportation^42^. The mobility index and bed utilization rate are visualized in Supplementary Fig. 5. We report the mean and standard deviation of county-level daily trips per person to be 3.49 and 0.88.

### Supplementary Information

Supplementary Figures.

## Data Availability

Publicly available datasets were analyzed in this study. This data can be found here: all data are cited.

## References

[1] Spiegel M, Tookes H (2021). Business restrictions and covid-19 fatalities. Rev. Financ. Stud..

[2] Hsiang S (2020). The effect of large-scale anti-contagion policies on the covid-19 pandemic. Nature.

[3] Singh S, Shaikh M, Hauck K, Miraldo M (2021). Impacts of introducing and lifting nonpharmaceutical interventions on covid-19 daily growth rate and compliance in the united states. Proc. Natl. Acad. Sci..

[4] Zhong, L., Diagne, M., Wang, Q. & Gao, J. Vaccination and three non-pharmaceutical interventions determine the dynamics of covid-19 in the us. *Hum. Soc. Sci. Commun.* **9** (2022).

[5] Mitjà O (2020). Experts’ request to the spanish government: Move spain towards complete lockdown. Lancet.

[6] Nasreen, S. *et al.* Effectiveness of covid-19 vaccines against symptomatic sars-cov-2 infection and severe outcomes with variants of concern in ontario. *Nat. Microbiol.* 1–7 (2022). 10.1038/s41564-021-01053-0 35132198

[7] Pouwels KB (2021). Effect of delta variant on viral burden and vaccine effectiveness against new sars-cov-2 infections in the UK. Nat. Med..

[8] Christie A (2021). Guidance for implementing covid-19 prevention strategies in the context of varying community transmission levels and vaccination coverage. Morb. Mortal. Wkly Rep..

[9] Moline HL (2021). Effectiveness of covid-19 vaccines in preventing hospitalization among adults aged $$\ge $$ 65 years-covid-net, 13 states, february-april 2021. Morb. Mortal. Wkly Rep..

[10] Wang, X., Wu, H. & Tang, S. Assessing age-specific vaccination strategies and post-vaccination reopening policies for covid-19 control using SEIR modeling approach. *medRxiv* (2021). 10.1007/s11538-022-01064-w PMC9418661 36029391

[11] Alagoz O (2021). The impact of vaccination to control covid-19 burden in the United States: A simulation modeling approach. PLoS ONE.

[12] Correa-Agudelo E, Mersha TB, Branscum AJ, MacKinnon NJ, Cuadros DF (2021). Identification of vulnerable populations and areas at higher risk of covid-19-related mortality during the early stage of the epidemic in the united states. Int. J. Environ. Res. Public Health.

[13] Cannistraci CV, Valsecchi MG, Capua I (2021). Age-sex population adjusted analysis of disease severity in epidemics as a tool to devise public health policies for covid-19. Sci. Rep..

[14] Ling L, Qian X, Guo S, Ukkusuri SV (2022). Spatiotemporal impacts of human activities and socio-demographics during the covid-19 outbreak in the us. BMC Public Health.

[15] Ling, L., Zhang, W., Bao, J. & Ukkusuri, S. V. Influencing factors for right turn lane crash frequency based on geographically and temporally weighted regression models. *J. Saf. Res.* (2023). 10.1016/j.jsr.2023.05.010 37718046

[16] Immunization, N. C. *et al.* Science brief: Community use of cloth masks to control the spread of sars-cov-2, in *CDC COVID-19 Science Briefs [Internet]*. (Centers for Disease Control and Prevention, 2021). 34009773

[17] Brüssow H, Zuber S (2022). Can a combination of vaccination and face mask wearing contain the covid-19 pandemic?. Microb. Biotechnol..

[18] Palcu J, Schreier M, Janiszewski C (2022). Facial mask personalization encourages facial mask wearing in times of covid-19. Sci. Rep..

[19] Chen, J. *et al.* Prioritizing allocation of covid-19 vaccines based on social contacts increases vaccination effectiveness. *medRxiv* (2021).

[20] Yabe T (2020). Non-compulsory measures sufficiently reduced human mobility in Tokyo during the covid-19 epidemic. Sci. Rep..

[21] Ling L, Murray-Tuite P, Lee S, Ge YG, Ukkusuri SV (2021). Role of uncertainty and social networks on shadow evacuation and non-compliance behavior in hurricanes. Transp. Res. Rec..

[22] Harvey, A. Trend analysis. *Wiley StatsRef Stat. Ref. Online* 1–21 (2014).

[23] Cooke, K. L. Differential-difference equations, in *International Symposium on Nonlinear Differential Equations and Nonlinear Mechanics*, 155–171 (Elsevier, 1963).

[24] Imbens, G. W. & Rubin, D. B. Rubin causal model, in *Microeconometrics*, 229–241 (Springer, 2010).

[25] Hunter, P. R. & Brainard, J. S. Estimating the effectiveness of the pfizer covid-19 bnt162b2 vaccine after a single dose. a reanalysis of a study of’real-world’vaccination outcomes from Israel. *Medrxiv* (2021).

[26] Matrajt L, Eaton J, Leung T, Brown ER (2021). Vaccine optimization for covid-19: Who to vaccinate first?. Sci. Adv..

[27] Freedman DO, Wilder-Smith A (2020). In-flight transmission of sars-cov-2: A review of the attack rates and available data on the efficacy of face masks. J. Travel Med..

[28] Barasheed O (2016). Uptake and effectiveness of facemask against respiratory infections at mass gatherings: A systematic review. Int. J. Infect. Dis..

[29] U.S., F. A. A. Airport capacity profiles. *Federal Aviat. Adm.* https://www.faa.gov/airports/planning_capacity/profiles/ (2021).

[30] Painter EM (2021). Demographic characteristics of persons vaccinated during the first month of the covid-19 vaccination program-united states, December 14, 2020-January 14, 2021. Morb. Mortal. Wkly Rep..

[31] Krishna K, Murty MN (1999). Genetic k-means algorithm. IEEE Trans. Syst. Man Cybern. Part B (Cybernetics).

[32] Yabe T, Zhang Y, Ukkusuri SV (2020). Quantifying the economic impact of disasters on businesses using human mobility data: A bayesian causal inference approach. EPJ Data Sci..

[33] Nakajima J, West M (2013). Bayesian analysis of latent threshold dynamic models. J. Bus. Econ. Stat..

[34] Scott SL, Varian HR (2014). Predicting the present with bayesian structural time series. Int. J. Math. Model. Numer. Opt..

[35] Brodersen KH, Gallusser F, Koehler J, Remy N, Scott SL (2015). Inferring causal impact using bayesian structural time-series models. Ann. Appl. Stat..

[36] Brandt PT, Freeman JR, Schrodt PA (2011). Real time, time series forecasting of inter-and intra-state political conflict. Confl. Manag. Peace Sci..

[37] Poyser O (2019). Exploring the dynamics of bitcoin’s price: A bayesian structural time series approach. Eurasian Econ. Rev..

[38] Gelman A, Jakulin A, Pittau MG, Su Y-S (2008). A weakly informative default prior distribution for logistic and other regression models. Ann. Appl. Stat..

[39] US, C. B. TIGER/Line geodatabases. *U. S. Census Bureau* https://www.census.gov/geographies/mapping-files/time-series/geo/tiger-geodatabase-file.html (2016).

[40] HHS. Hospital Utilization. *Health Hum. Serv. (HHS) Prot. Publ. Data Hub* https://protect-public.hhs.gov/pages/hospital-utilization (2020).

[41] Foundation, K. F. State covid-19 data and policy actions. *KFF* https://www.kff.org/coronavirus-covid-19/issue-brief/state-covid-19-data-and-policy-actions/ (2020).

[42] US, D. Distribution of trips by distance: National, state, and county level. *US Dep. Transp.* https://www.bts.gov/browse-statistical-products-and-data/covid-related/distribution-trips-distance-national-state-and (2020).

[43] You C (2020). Estimation of the time-varying reproduction number of covid-19 outbreak in China. Int. J. Hyg. Environ. Health.

[44] Shi, A., Gaynor, S. M., Quick, C. & Lin, X. Multi-resolution characterization of the covid-19 pandemic: A unified framework and open-source tool. *medRxiv* (2021).

[45] CDC. Reporting county-level covid-19 vaccination data. *Cent. Dis. Control Prev.* https://www.cdc.gov/coronavirus/2019-ncov/vaccines/distributing/reporting-counties.html (2021).

[46] Wellenius GA (2021). Impacts of social distancing policies on mobility and covid-19 case growth in the us. Nat. Commun..

[47] Lucchini L (2021). Living in a pandemic: changes in mobility routines, social activity and adherence to covid-19 protective measures. Sci. Rep..

