# Supplementary material for: Investigating the effects of vaccine on COVID-19 disease propagation using a Bayesian approach
Source: Sci Rep. 2023 Aug 17;13:13374. doi: 10.1038/s41598-023-37972-7 (PMC10435512; doi:10.1038/s41598-023-37972-7)

Supplementary material

Supplementary Figure 1: **BSTS model training, testing, and prediction for Potter county in Texas**. (A) BSTS model prediction (predicted value of $\widehat{R(t)}_{i,t,f}$ and the observed value of $R(t)_{i,t,f}$. (B) Estimated causal impact $\theta_{i,t,f}$. f refers to the Potter county in Texas. The blue area refers to the period of model training, the yellow area refers to the period of model testing, and the rest is the $R(t)$ prediction.

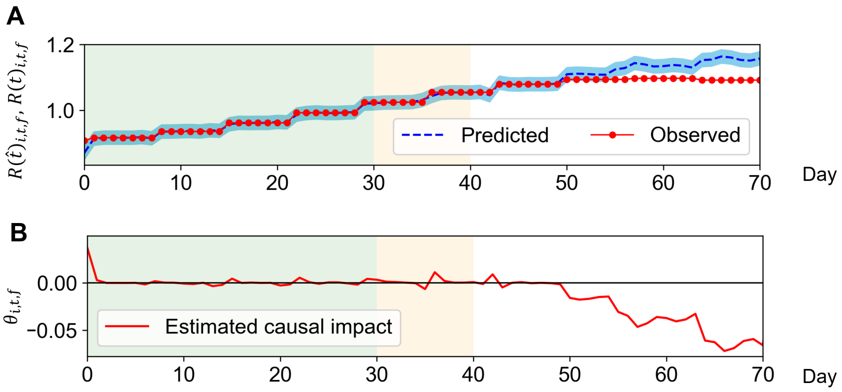

Supplementary Figure 2: **Model calibration**. (A) Sensitivity of hyperparameters of prior distribution, and (B) Q-Q plot examining the normality assumption of the residuals.

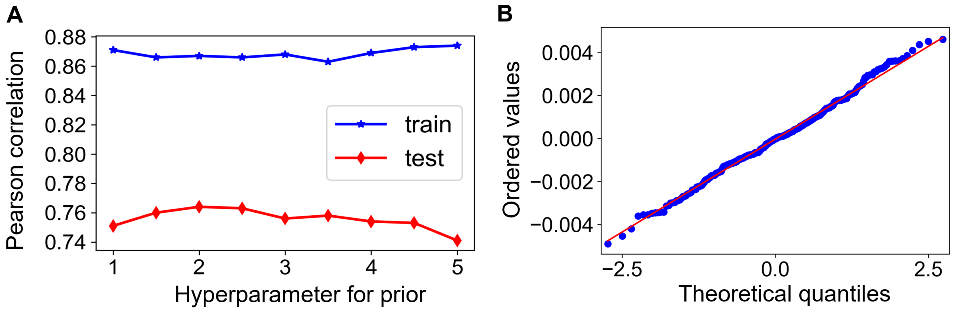

Supplementary Figure 3: **Temporal variation of the disease propagation and vaccine coverage**. (A)Effective reproduction number $R(t)$. (B) Vaccine coverage. The mean of $R(t)$ for 'class1', 'class2', 'class3', and 'airport' are 1.15, 1.11, 1.25, and 1.20; the mean of vaccine coverage for 'class1', 'class2', 'class3', and 'airport' are 16.44, 13.27, 16.99, and 15.83.

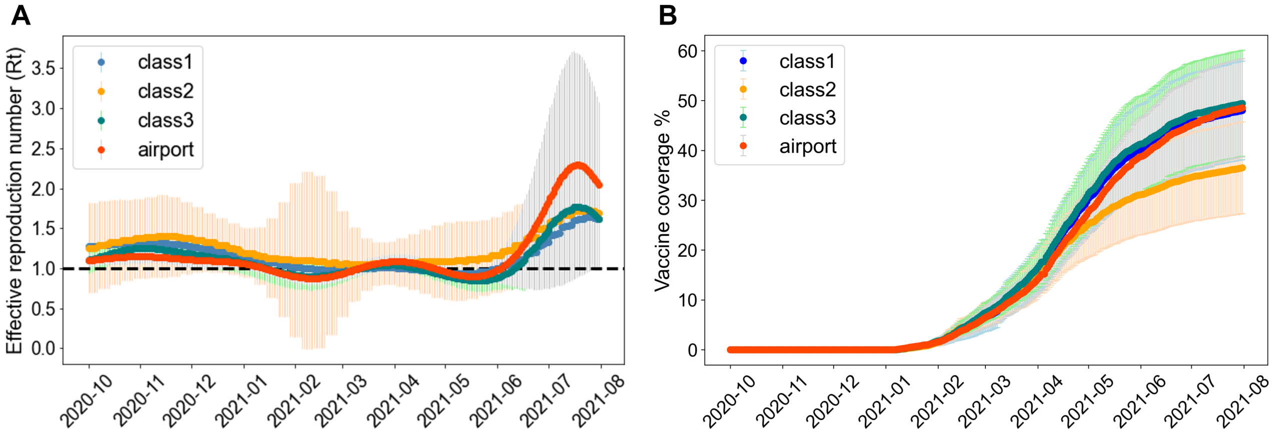

Supplementary Figure 4: **Relaxing procedure of the state-level NPIs and the presence of the Delta: business reopening, mask requirement, and the presence of the Delta variant**. It is clear that the $R(t)$ surged after two-weeks of the Delta detection for almost all sample states. Therefore, we select two-weeks

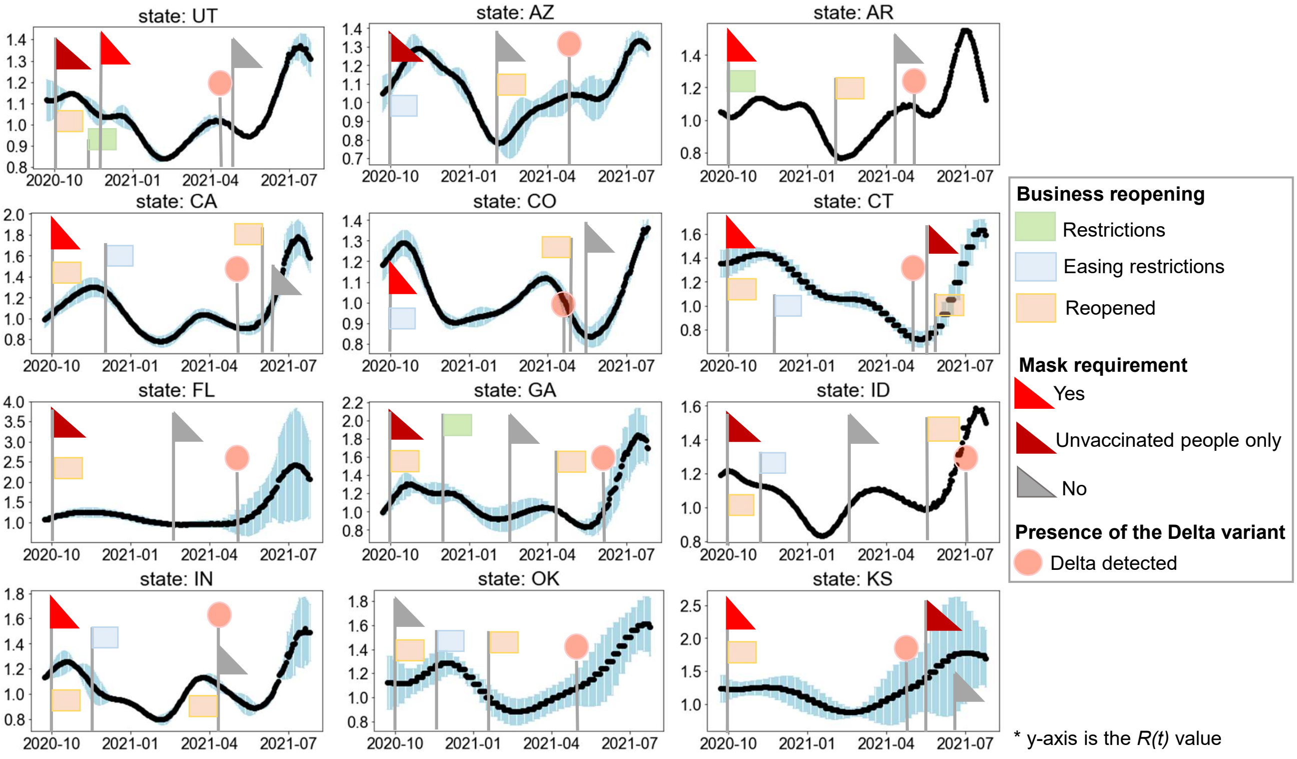

Supplementary Figure 5: **The time variation of mobility index and bed utilization rate within four county classes**. (A) Mobility index (daily trip per person). (B) Average daily hospital bed utilization %.

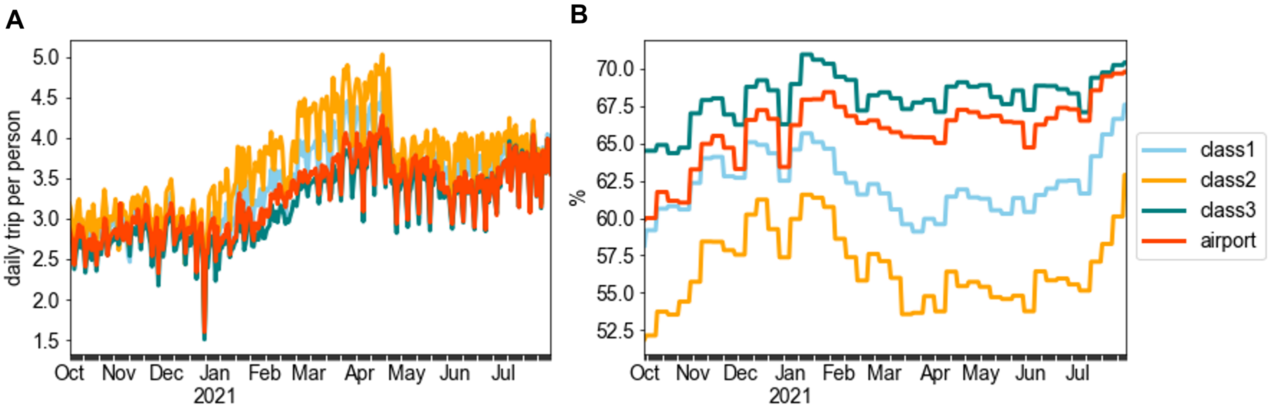

Supplement: Supplementary file 1 — Supplementary Figures. [file 41598_2023_37972_MOESM1_ESM.pdf]
